# Anatomical site and histological subtype as determinants of Breslow thickness and high-risk features in surgically managed cutaneous melanoma at Charlotte Maxeke Johannesburg Academic Hospital, Johannesburg, South Africa

**DOI:** 10.3389/fsurg.2026.1826225

**Published:** 2026-08-25

**Authors:** Rahmiz Thomas, Yazeerah Bhana, Marina Butoi, Inam Madikizela, Sibusiso Mahlangu, Zoë Malan, Thato Mpapa, Ethan Naidoo, Wilhelm Hansen, Thifhelimbilu Emmanuel Luvhengo, Nosisa Thabile Sishuba

**Affiliations:** 1 Department of Surgery, Charlotte Maxeke Johannesburg Academic Hospital, Johannesburg, South Africa; 2 Department of Surgery, School of Clinical Medicine, Faculty of Health Sciences, University of the Witwatersrand, Johannesburg, South Africa; 3 Independent Researcher, Johannesburg, South Africa

**Keywords:** acral lentiginous melanoma, Breslow thickness, melanoma, nodular melanoma, South Africa

## Abstract

**Background:**

Breslow thickness is central to staging and prognosis in cutaneous melanoma and informs decisions on excision and nodal staging. We evaluated whether anatomical site and histological subtype predict Breslow thickness and selected high-risk pathological features in a surgically managed South African cohort, and whether Breslow thickness was associated with ulceration, mitotic rate, and nodal positivity.

**Methods:**

We performed a retrospective observational study of surgically managed primary cutaneous melanoma at Charlotte Maxeke Johannesburg Academic Hospital, Johannesburg, South Africa, from January 2016 to December 2023. Breslow thickness was right-skewed and was summarised using medians with interquartile ranges. Categorical variables were summarised using counts and percentages. Adjusted associations with Breslow thickness were examined using multivariable linear regression on log-transformed Breslow thickness. Adjusted associations with ulceration and nodal positivity were examined using multivariable logistic regression.

**Results:**

Ninety six melanomas were included. Breslow thickness was recorded in 88 cases, of which 86 had positive Breslow values for Breslow-based analyses. In the adjusted anatomical site model (*n* = 85), acral melanomas were thicker than trunk melanomas [ratio 2.32, 95% confidence interval (CI) 1.17–4.58, *p* = 0.016], and lower limb melanomas were also thicker than trunk melanomas (ratio 1.83, 95% CI 1.08–3.11, *p* = 0.025). In the adjusted subtype model (*n* = 72), acral lentiginous melanoma (ratio 3.49, 95% CI 1.86–6.56, *p* < 0.001) and nodular melanoma (ratio 2.92, 95% CI 1.60–5.35, *p* < 0.001) were thicker than superficial spreading melanoma. Lentigo maligna melanoma was also thicker than superficial spreading melanoma (ratio 3.52, 95% CI 1.93–6.40, *p* < 0.001), although this estimate was based on only five cases. Increasing Breslow thickness was associated with ulceration (odds ratio 2.90, 95% CI 1.67–5.04, *p* < 0.001), whereas neither Breslow thickness nor ulceration was independently associated with nodal positivity.

**Conclusion:**

Anatomical site and histological subtype identify recognisable groups at risk of presenting with thick melanoma. These findings support earlier biopsy and expedited surgical referral for acral, lower limb, and nodular appearing lesions in public sector pathways. Larger prospective studies with follow up data are needed to determine whether these patterns translate into differences in oncological outcomes

## Introduction

Cutaneous melanoma accounts for a disproportionate share of skin cancer mortality because survival declines steeply with increasing tumour thickness and stage at diagnosis (1).

Contemporary global estimates continue to show a substantial and uneven melanoma burden, which reinforces the need for context-specific data to guide service planning in resource-constrained systems (2). Although melanoma is less common than many other malignancies, its prognosis is highly sensitive to diagnostic timing. For this reason, factors associated with thicker disease at presentation remain clinically important.

In South Africa, registry-based analyses have shown marked differences across population groups. Melanoma is less common in Black Africans, but is more likely to involve the limbs and to present at greater thickness, which is consistent with delayed diagnosis and more limited access to early specialist assessment (3, 4). Acral melanoma is particularly relevant in populations with skin of colour because lesions on the palms, soles, and nail apparatus are frequently concealed, misattributed to benign or traumatic causes, or referred late (5, 6). These patterns make local clinicopathological data especially important in the South African public sector context.

Breslow thickness remains central to contemporary melanoma staging and directly informs surgical management, including excision margins and nodal staging decisions (1, 7). Histological subtype also carries clinically meaningful information. Nodular melanoma is characterised by early vertical growth and often presents with thicker disease, whereas superficial spreading melanoma is more often detected at thinner stages (8, 9). Risk factor meta-analysis also supports the view that anatomical site and clinico-pathological variant represent distinct but overlapping melanoma phenotypes (10).

In the South African public sector, delayed progression from biopsy to definitive surgery has already been documented as a modifiable pathway problem (11). For surgically managed melanoma in this setting, identifying readily recognisable clinicopathological flags for thick presentation may therefore be practically useful. We aimed to evaluate whether anatomical site and histological subtype predict Breslow thickness and selected high-risk pathological features, namely ulceration, mitotic rate, and nodal metastasis, in a surgically managed cohort at Charlotte Maxeke Johannesburg Academic Hospital. We further aimed to explore whether increasing Breslow thickness was associated with ulceration and nodal positivity in this dataset.

## Materials and methods

### Study design and setting

This was a single-centre retrospective observational study of surgically managed primary cutaneous melanomas treated at Charlotte Maxeke Johannesburg Academic Hospital (CMJAH), Johannesburg, South Africa, between 1 January 2016 and 31 December 2023. The study focused on surgically managed primary cutaneous melanoma cases within the public sector pathway at this tertiary academic centre. Reporting follows the Strengthening the Reporting of Observational Studies in Epidemiology (STROBE) statement. The study was designed to evaluate clinicopathological associations at presentation rather than long-term oncological outcomes.

### Participants

Patients with histologically confirmed primary cutaneous melanoma managed surgically during the study period were eligible. Cases were retained for descriptive reporting where key variables were missing. Complete-case analyses were performed for each model. Two cases had Breslow values recorded as 0.0 mm and corresponded to T0/Tx-stage entries rather than measurable invasive primary tumour thickness; these cases were retained in descriptive cohort reporting but excluded from Breslow-based regression analyses.

### Variables and outcomes

The primary outcome was Breslow thickness (mm). Secondary outcomes were ulceration, mitotic rate, and nodal metastasis. Anatomical sites were categorised as head and neck, upper limb, trunk, lower limb, acral, and mixed, where multiple anatomical sites were recorded. Histological subtype was grouped as acral lentiginous, nodular, superficial spreading, and lentigo maligna melanoma, with rarer categories retained descriptively but excluded from the primary subtype model.

Nodal status was based solely on pathological staging, as documented in the pathology-linked clinical record. Where the treatment field allowed further classification, nodal assessment was categorised as sentinel lymph node biopsy, therapeutic lymph node dissection, or lymph node biopsy. Among 94 cases with documented nodal status, 59 had sentinel lymph node biopsy documented, 10 had therapeutic lymph node dissection documented, 1 had lymph node biopsy documented, and in 24 the exact pathological nodal procedure was not explicitly specified in the treatment field.

### Statistical analysis

Continuous variables were assessed for distributional shape before analysis. Breslow thickness showed a right-skewed distribution, and continuous variables were therefore summarised using medians with interquartile ranges, while categorical variables were summarised as counts and percentages. Because Breslow thickness was right-skewed, adjusted associations with Breslow thickness were examined using multivariable linear regression on log-transformed Breslow thickness. Two cases had Breslow values recorded as 0.0 mm and corresponded to T0/Tx-stage entries rather than measurable invasive primary tumour thickness; these cases were retained in descriptive reporting but excluded from Breslow-based regression analyses. Because the assumptions for parametric testing were not fulfilled for Breslow thickness, non-parametric methods were used for unadjusted comparisons. Breslow thickness was compared across anatomical sites and histological subtypes using the Kruskal–Wallis test with Dunn *post hoc* pairwise comparisons and false discovery rate adjustment. Associations between Breslow thickness and ulceration and between mitotic rate and ulceration were assessed using the Mann–Whitney *U*-test. Adjusted associations with ulceration and nodal positivity were examined using multivariable logistic regression. Associations between ulceration and nodal positivity were explored in an unadjusted analysis using odds ratios and Fisher's exact test. A two-sided *p* < 0.05 was considered statistically significant. Race/population group was summarised descriptively. Because categories outside White and Black were sparse, race was not included in the primary multivariable models to avoid unstable estimates, but was explored in a parsimonious sensitivity analysis using White vs. Black categorisation.

### Ethics

Ethics approval for this retrospective study was obtained from the Human Research Ethics Committee (HREC), approval number M241046. The requirement for informed consent was waived in accordance with institutional policy for retrospective record review. Standardised pathology reporting elements align with contemporary College of American Pathologists (CAP) melanoma protocols and World Health Organization (WHO) skin tumour classification frameworks. All data were handled in accordance with institutional confidentiality requirements.

## Results

### Cohort overview

**Figure 1:**
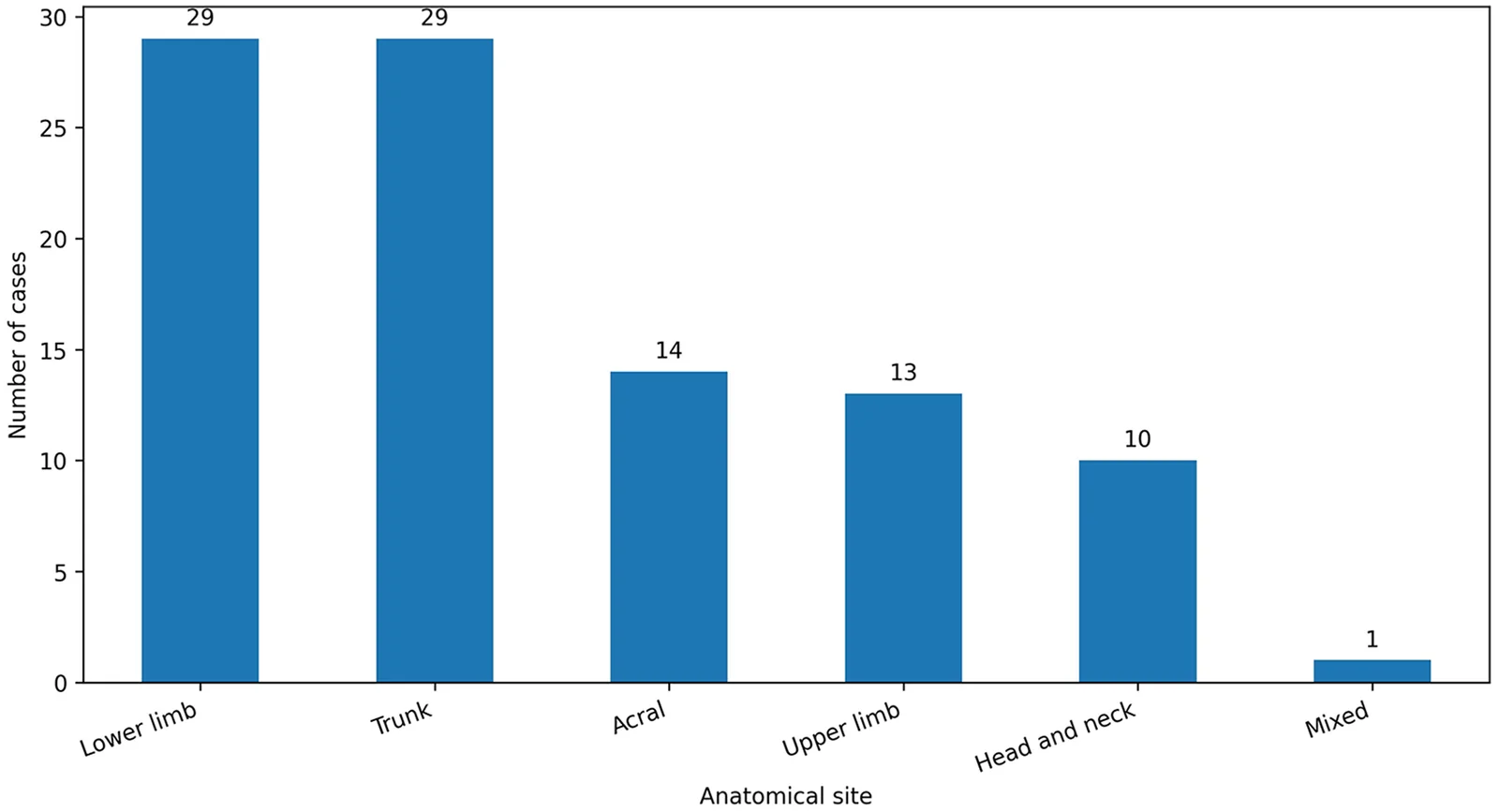
Distribution of primary melanoma by anatomical site (*n* = 96).

Ninety-six surgically managed melanomas were included (Table 1 and Figure 1). The median age was 61.5 years (IQR 52.0–71.2). Fifty patients (52.1%) were female and 46 (47.9%) were male.

**Table 1:** Cohort characteristics (*n* = 96).

**Characteristic**	**Value**
Total included, *n*	96
Age, years, median (IQR)	61.5 (52.0–71.2)
Sex, *n* (%)	
Female	50 (52.1)
Male	46 (47.9)
Race/population group, *n* (%)	
White	65 (67.7)
Black	29 (30.2)
Asian	1 (1.0)
Missing	1 (1.0)
Anatomical site, *n* (%)	
Lower limb	29 (30.2)
Trunk	29 (30.2)
Acral	14 (14.6)
Upper limb	13 (13.5)
Head and neck	10 (10.4)
Mixed	1 (1.0)
Breslow thickness recorded, *n* (%)	88 (91.7)
Positive Breslow values for Breslow-based analyses, *n* (%)	86 (89.6)
Positive Breslow thickness, median (IQR), mm	4.00 (2.00–8.00)
Histological subtype recorded, *n* (%)	81 (84.4)
Ulceration documented, *n* (%)	83 (86.5)
Mitotic rate documented, *n* (%)	67 (69.8)
Nodal status documented, *n* (%)	94 (97.9)

IQR, interquartile range. Two cases with Breslow values recorded as 0.0 mm corresponded to T0/Tx-stage entries and were excluded from Breslow-based analyses.

Race/population group was documented as White in 65 cases (67.7%), Black in 29 (30.2%), Asian in 1 (1.0%), and missing in 1 (1.0%). Breslow thickness was recorded in 88 cases. After exclusion of two 0.0 mm T0/Tx entries, 86 cases had positive Breslow values for Breslow-based analyses. Histological subtype was recorded in 81 cases, ulceration in 83, mitotic rate in 67, and nodal status in 94. The median positive Breslow thickness in the cohort was 4.00 mm (IQR 2.00–8.00).

### Anatomical site and Breslow thickness

**Figure 2:**
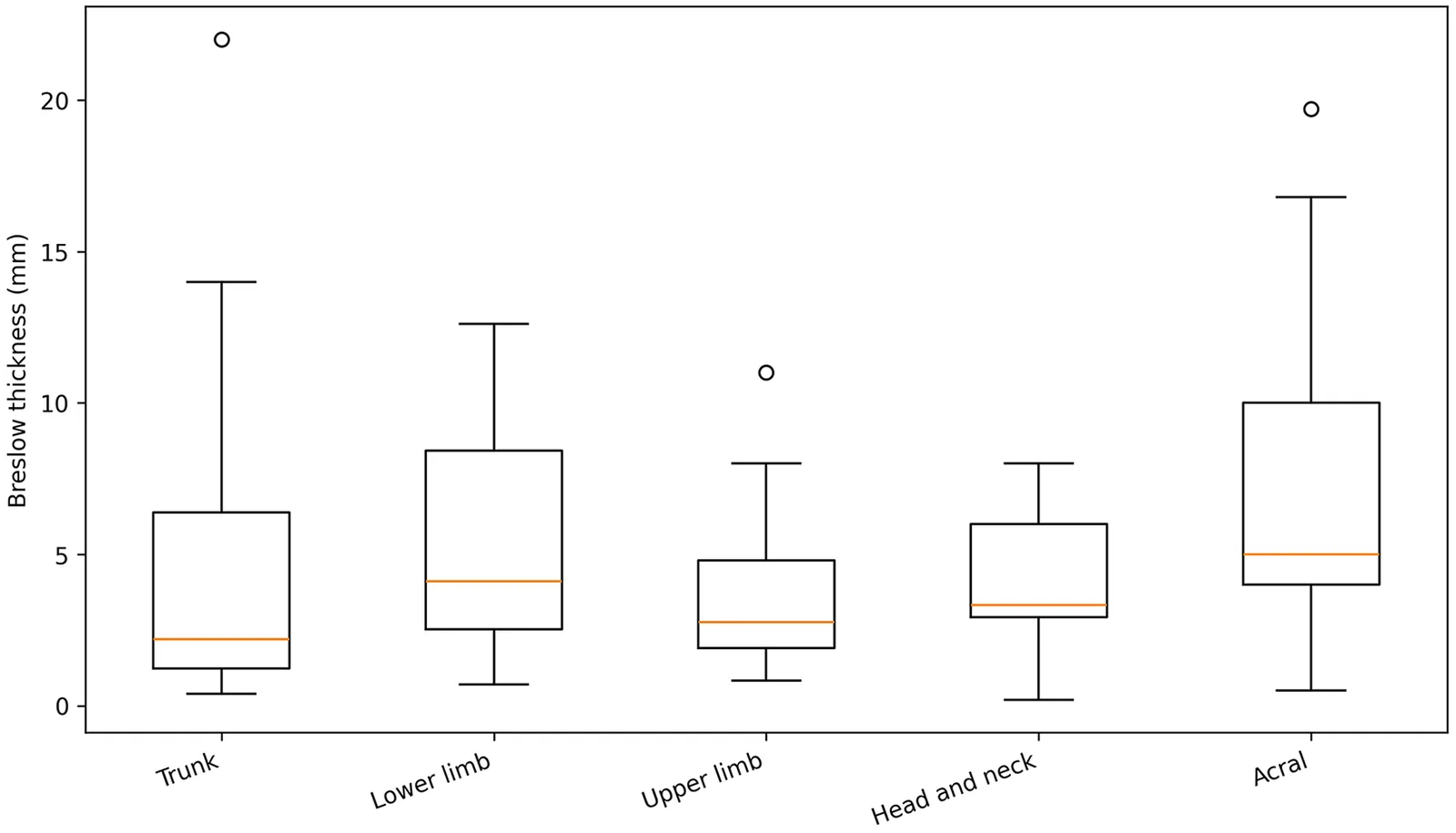
Breslow thickness by anatomical site among cases with positive breslow values and non-mixed site coding (*n* = 85). Box-and-whisker plots show the median, interquartile range, whiskers, and outlier points.

Among cases with positive Breslow values and non-mixed site coding, acral melanomas had the highest median Breslow thickness, followed by lower limb melanomas (Table 2 and Figure 2). In the adjusted site model (*n* = 85), acral melanomas were thicker than trunk melanomas (ratio 2.318, 95% CI 1.167–4.606, *p* = 0.016), and lower limb melanomas were also thicker than trunk melanomas (ratio 1.829, 95% CI 1.078–3.104, *p* = 0.025) (Table 3). In sensitivity analyses additionally adjusting for race as White vs. Black, the direction of the primary site associations was preserved, although the acral estimate attenuated with wider confidence intervals.

**Table 2:** Breslow thickness by anatomical site and histological subtype among cases with positive Breslow values.

**Group**	**n**	**Median Breslow (mm) [IQR]**
Anatomical site		
Trunk	26	2.20 [1.23–6.38]
Lower limb	28	4.10 [2.53–8.43]
Upper limb	10	2.75 [1.90–4.80]
Head and neck	8	3.33 [2.93–6.00]
Acral	13	5.00 [4.00–10.00]
Histological subtype (main cutaneous subtypes)		
Superficial spreading	13	1.84 [0.88–2.40]
Lentigo maligna	5	6.00 [3.36–6.30]
Nodular	29	5.00 [2.10–8.40]
Acral lentiginous	25	4.90 [3.00–9.50]

**Table 3:** Model A: log(Breslow)∼site + age + sex (reference: trunk); positive breslow values only, *n* = 85.

**Predictor**	**Estimate**	**95% CI low**	**95% CI high**	***p* value**
Lower limb vs. trunk	1.829	1.078	3.104	0.025
Upper limb vs. trunk	1.367	0.690	2.706	0.370
Head and neck vs. trunk	1.239	0.512	2.999	0.635
Acral vs. trunk	2.318	1.167	4.606	0.016
Age (per year)	1.003	0.987	1.020	0.682
Male vs. female	1.574	1.026	2.414	0.038

### Histological subtype and Breslow thickness

**Figure 3:**
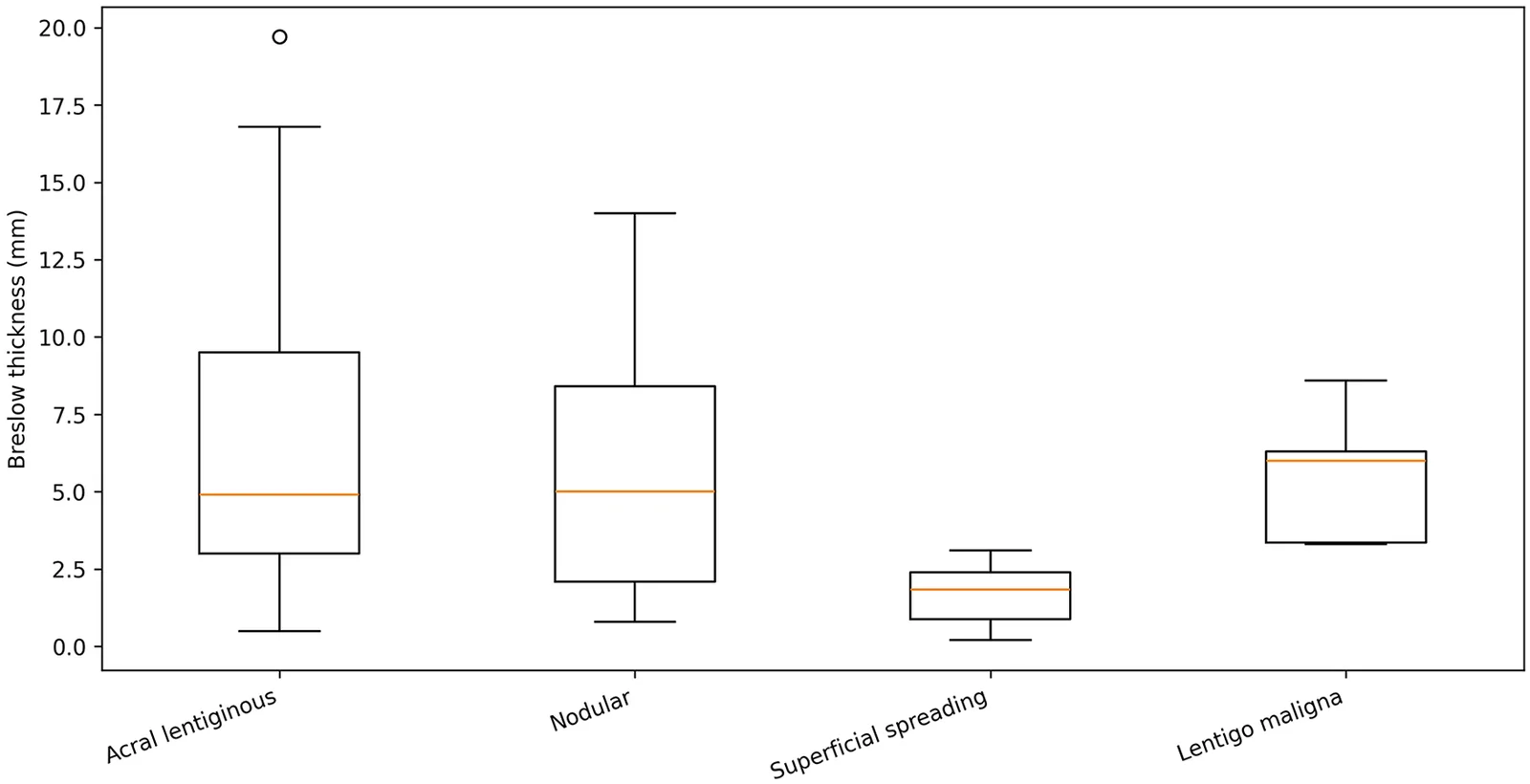
Breslow thickness by histological subtype among the four main cutaneous subtypes with positive breslow values (*n* = 72). Box-and-whisker plots show the median, the interquartile range, the whiskers, and outliers.

Among the four main cutaneous subtypes with positive Breslow values available (*n* = 72), median Breslow thickness was greatest for lentigo maligna melanoma, acral lentiginous melanoma, and nodular melanoma, and lowest for superficial spreading melanoma (Table 2 and Figure 3). In the adjusted subtype model, nodular melanoma (ratio 2.922, 95% CI 1.573–5.426, *p* < 0.001), acral lentiginous melanoma (ratio 3.494, 95% CI 1.868–6.533, *p* < 0.001), and lentigo maligna melanoma (ratio 3.516, 95% CI 1.900–6.507, *p* < 0.001) were thicker than superficial spreading melanoma (Table 4A). The lentigo maligna estimate should, however, be interpreted cautiously because it was based on only five cases.

**Table 4A:** Model B: log(Breslow) ∼ subtype + age + sex (reference: superficial spreading); positive breslow values only, *n* = 72.

**Predictor**	**Estimate**	**95% CI low**	**95% CI high**	***p* value**
Lentigo maligna vs. superficial spreading	3.516	1.900	6.507	<0.001
Nodular vs. superficial spreading	2.922	1.573	5.426	<0.001
Acral lentiginous vs. superficial spreading	3.494	1.868	6.533	<0.001
Age (per year)	1.006	0.987	1.026	0.517
Male vs. female	1.343	0.917	1.965	0.130

### Ulceration, mitotic rate, and nodal status

**Figure 4:**
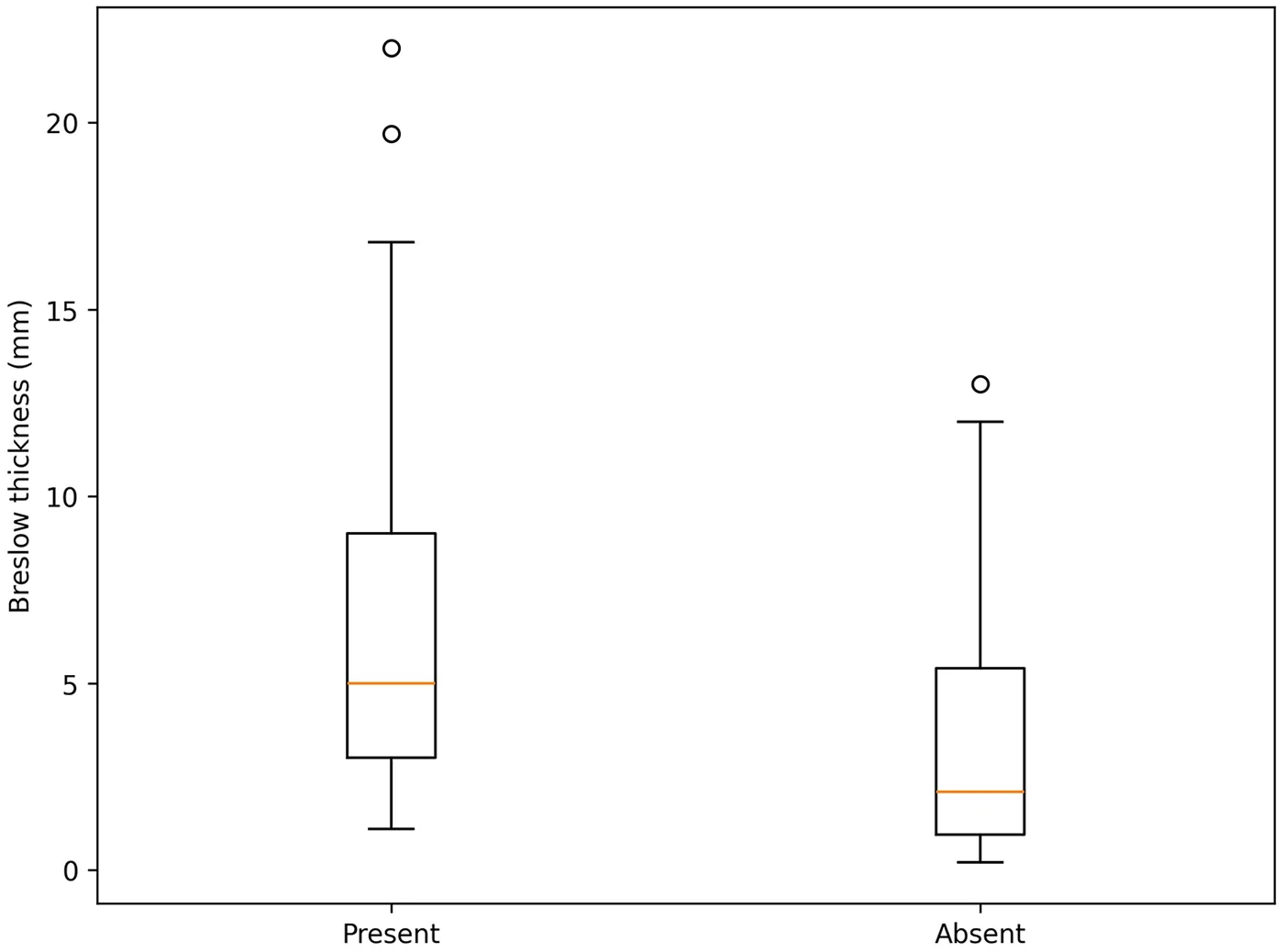
Breslow thickness by ulceration status among cases with positive Breslow values and documented ulceration (*n* = 80). Box-and-whisker plots show the median, interquartile range, whiskers, and outliers.

**Figure 5:**
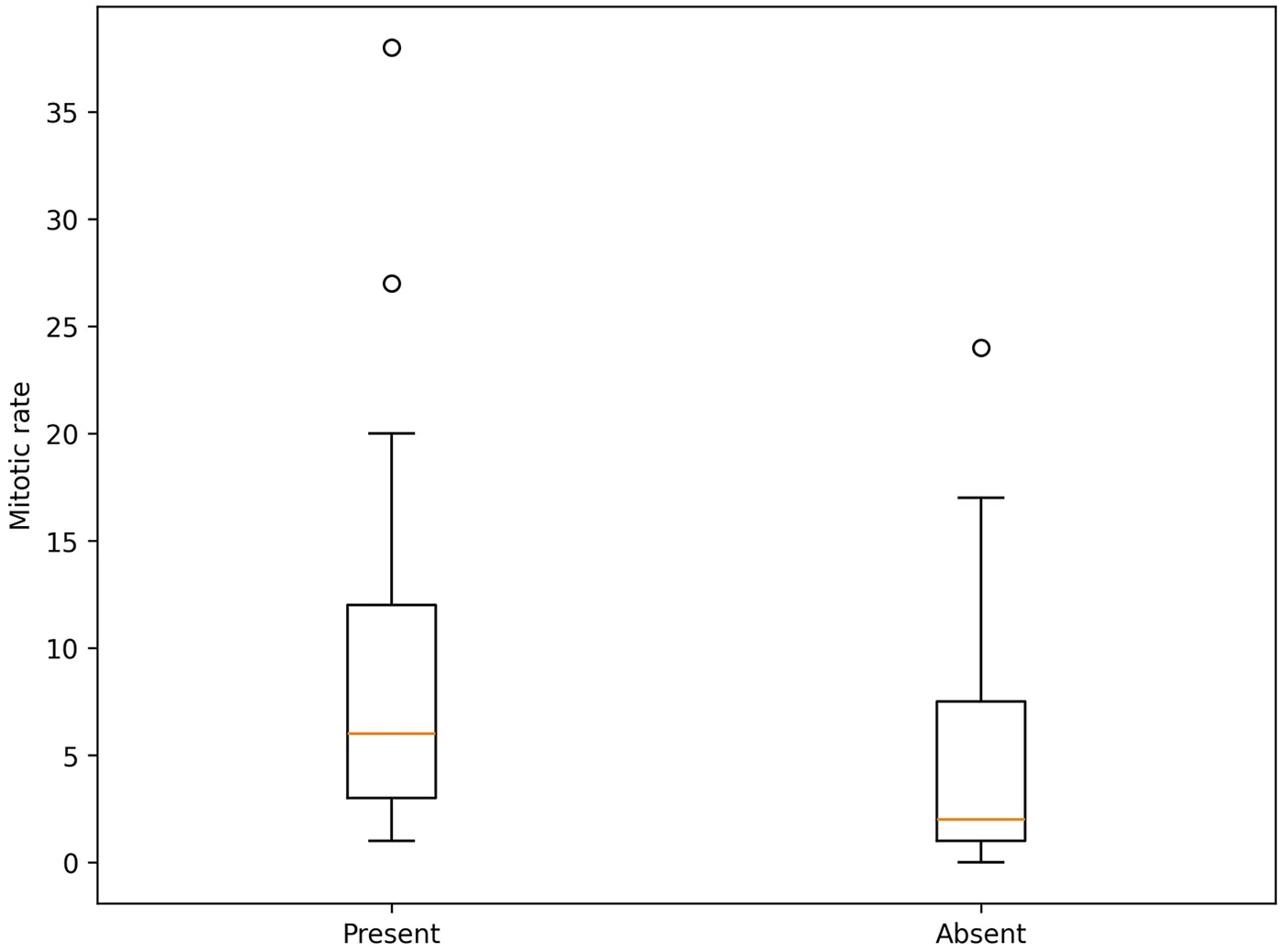
Mitotic rate by ulceration status (*n* = 67). Box-and-whisker plots show the median, interquartile range, whiskers, and outlier points.

Among cases with positive Breslow values and documented ulceration (*n* = 80), ulcerated tumours had greater Breslow thickness than non-ulcerated tumours [median 5.00 mm [IQR 3.00–9.00] vs. 2.10 mm [IQR 0.95–5.40], *p* < 0.001] (Table 4B and Figure 4). Mitotic rate was also higher in ulcerated tumours [median 6.0/mm^2^ [IQR 3.0–12.0] vs. 2.0/mm^2^ [IQR 1.0–7.5], *p* = 0.004]. In the adjusted ulceration model (*n* = 80), increasing Breslow thickness was associated with ulceration (OR 2.899, 95% CI 1.666–5.044, *p* < 0.001) (Table 4C and Figure 5). These findings are consistent with ulceration behaving as an adverse pathological feature in thicker tumours.

**Table 4B:** Unadjusted associations.

Comparison	*N*	Group 1	Group 2	Effect/*p* value
Breslow thickness by ulceration	80	Ulcerated, *n* = 45, 5.00 [3.00–9.00]	Non-ulcerated, *n* = 35, 2.10 [0.95–5.40]	*p* < 0.001
Mitotic rate by ulceration	67	Ulcerated, *n* = 40, 6.0 [3.0–12.0]	Non-ulcerated, *n* = 27, 2.0 [1.0–7.5]	*p* = 0.004
Ulceration vs. nodal positivity	82	Ulcerated vs. non-ulcerated		OR 1.40,
				*p* = 0.508

**Table 4C:** Model C: ulceration (present) ∼ log(Breslow) + age + sex; positive Breslow values only, *n* = 80.

**Predictor**	**Estimate**	**95% CI low**	**95% CI high**	***p* value**
log(Breslow)	2.899	1.666	5.044	<0.001
Age (per year)	0.984	0.952	1.017	0.344
Male vs. female	1.860	0.673	5.142	0.231

Among cases with both documented nodal status and ulceration (*n* = 82), ulceration was not associated with nodal positivity on unadjusted analysis (OR 1.40, *p* = 0.508). In the adjusted nodal model restricted to cases with positive Breslow values (*n* = 80), neither Breslow thickness (OR 1.625, 95% CI 0.936–2.821, *p* = 0.085) nor ulceration (OR 0.891, 95% CI 0.314–2.527, *p* = 0.828) was independently associated with nodal positivity (Table 4D). Although the point estimate for Breslow thickness trended upward, the confidence interval crossed unity. This suggests either limited power, heterogeneity in the nodal pathway, or both.

**Table 4D:** Model D: nodal positivity ∼ log(Breslow) + ulceration + age + sex; positive breslow values only, *n* = 80.

**Predictor**	**Estimate**	**95% CI low**	**95% CI high**	***p* value**
log(Breslow)	1.625	0.936	2.821	0.085
Ulceration present vs. absent	0.891	0.314	2.527	0.828
Age (per year)	0.972	0.940	1.005	0.098
Male vs. female	0.799	0.316	2.017	0.634

## Discussion

In this single-centre South African surgical series, anatomical site and histological subtype were clinically meaningful determinants of Breslow thickness at presentation. After exclusion of two non-measurable 0.0 mm T0/Tx entries from Breslow-based modelling, both acral and lower limb lesions remained thicker than trunk melanomas in adjusted analyses, while acral lentiginous and nodular melanomas remained thicker than superficial spreading melanoma. These findings suggest that recognisable lesion patterns may help identify patients at risk of presenting with thicker disease.

These findings fit with the South African literature. National registry analyses have shown that melanoma in Black Africans is less common but more likely to involve the limbs and to present at greater thickness (3). South African acral series have also reported late presentation, high Breslow thickness, and advanced stage (5, 6). Within the public sector, delayed progression from diagnostic biopsy to definitive surgery has been documented elsewhere in South Africa (11). Together, these observations support the view that a meaningful component of thickness burden may be modifiable through earlier recognition and faster progression along the pathway.

The acral signal is clinically important, but it requires careful interpretation. The acral site signal and the acral lentiginous subtype signal should not be interpreted as wholly independent findings. Although we modelled site and subtype separately to reduce collinearity, these variables partly describe overlapping patient groups, one anatomically and the other histopathologically. The results are therefore best interpreted as complementary clinical lenses on a high-risk phenotype rather than as separate causal effects. This distinction is important when considering how the findings should inform clinical triage and pathway design.

The subtype findings are biologically plausible. Nodular melanoma is characterised by early vertical growth, and large registry analyses have shown that primary nodular melanoma is associated with poorer outcomes than other subtypes even after adjustment for thickness and stage (8). In the present cohort, the estimate of lentigo maligna was also large, but the subgroup was very small, and the coded cases were unexpectedly thick. We therefore present that estimate transparently while avoiding strong biological inference from it. The more robust signal in this dataset lies in the contrast between superficial spreading melanoma and the thicker acral lentiginous and nodular subtypes.

Ulceration behaved as expected for an adverse pathological feature. It tracked with greater thickness and higher mitotic activity, and increasing Breslow thickness remained associated with ulceration after adjustment. By contrast, we did not demonstrate an independent association between Breslow thickness or ulceration and nodal positivity in the adjusted nodal model. This may reflect limited power, variable staging pathways that were not fully recoverable from the retrospective record, or true biological heterogeneity. The absence of an independent association in this model should therefore be interpreted cautiously rather than as evidence of no relationship.

The findings have practical implications for surgical oncology pathways. Persistent acral or nail apparatus lesions should attract a low threshold for biopsy and expedited referral. Rapidly growing nodular-appearing lesions should likewise be prioritised because of their tendency toward early vertical growth. Public sector pathways should also prioritise synoptic melanoma pathology reporting and prospective auditing of the intervals from presentation to biopsy, biopsy to definitive excision, and excision to nodal staging**.** Such process-level auditing may be especially important in settings where service bottlenecks contribute to delayed treatment.

Contemporary guideline frameworks continue to support risk-adapted wide local excision and selective sentinel lymph node biopsy for appropriate primary tumours (7, 12, 13). Randomised evidence does not support routine completion lymph node dissection after a positive sentinel node for all patients, although immediate node dissection does improve regional control (14–16). For acral melanoma, contemporary reviews continue to emphasise distinct biology and relatively limited, heterogeneous outcome data in the adjuvant setting (17, 18). These broader evidence-based frameworks help contextualise the present findings within contemporary melanoma care. They also reinforce the importance of identifying high-risk presentations early, before patients enter more complex treatment pathways.

### Limitations

This was a single-centre retrospective study with incomplete data for several variables. No reliable follow-up or recurrence dataset was available, so survival analysis could not be performed. The study therefore addresses clinicopathological associations at presentation rather than longitudinal oncological outcomes. This distinction should be kept in mind when interpreting the practical implications of the findings.

Nodal status in this cohort was pathology-based, but the exact pathological nodal procedure was not explicitly specified in the treatment field for all cases. Residual confounding by population group remains possible. Although race was available descriptively and explored in sensitivity analyses, the small cohort and sparse non-White categories limited stable race adjustment in the primary models. Finally, small subgroup sizes, particularly for lentigo maligna melanoma, limit the precision of some estimates and warrant cautious interpretation. These limitations do not negate the descriptive and hypothesis-generating value of the study, but they do constrain causal inference.

## Conclusion

Anatomical site and histological subtype were associated with Breslow thickness in this surgically managed South African melanoma cohort. Acral and lower limb melanomas were thicker than trunk melanomas in adjusted analyses of positive Breslow values, and acral lentiginous, nodular, and small-number lentigo maligna cases were thicker than superficial spreading melanoma.

Increasing Breslow thickness was associated with ulceration, whereas neither Breslow thickness nor ulceration was independently associated with nodal positivity in this dataset. These findings support earlier biopsy, expedited referral, and pathway-level quality improvement efforts for recognisable high-risk melanoma presentations within the public sector.

## Data Availability

The raw data supporting the conclusions of this article will be made available by the authors, without undue reservation.
